# Supplementary material for: Resolving the full spectrum of human genome variation using Linked-Reads
Source: Genome Res. 2019 Apr;29(4):635–45. doi: 10.1101/gr.234443.118 (PMC6442396; doi:10.1101/gr.234443.118)
Supplement: Supplemental Material [file supp_gr.234443.118_Supplemental_longranger-2.2.zip › longranger-2.2.tar.bz2!/lr2.2-public/lib/stan/cmdstan/stan/doc/api/html/run__markov__chain_8hpp_source.html]

Stan: src/stan/common/run\_markov\_chain.hpp Source File


|  |  |
| --- | --- |
|  | Stan  2.5.0  probability, sampling & optimization |


- Main Page
- Related Pages
- Namespaces
- Classes
- Files

- File List
- File Members

All Classes Namespaces Files Functions Variables Typedefs Enumerations Enumerator Friends Macros Pages

- src
- stan
- common

run\_markov\_chain.hpp

Go to the documentation of this file.

1 #ifndef STAN\_\_COMMON\_\_RUN\_MARKOV\_CHAIN\_HPP

2 #define STAN\_\_COMMON\_\_RUN\_MARKOV\_CHAIN\_HPP

3

4 #include <stan/mcmc/base\_mcmc.hpp>

5 #include <stan/io/mcmc\_writer.hpp>

6 #include <stan/common/print\_progress.hpp>

7

8 namespace stan {

9  namespace common {

10

11  template <class Model, class RNG, class StartTransitionCallback,

12  class SampleRecorder, class DiagnosticRecorder, class MessageRecorder>

13  void run\_markov\_chain(stan::mcmc::base\_mcmc\* sampler,

14  const int num\_iterations,

15  const int start,

16  const int finish,

17  const int num\_thin,

18  const int refresh,

19  const bool save,

20  const bool warmup,

21  stan::io::mcmc\_writer <Model,

22  SampleRecorder, DiagnosticRecorder, MessageRecorder>&

23  writer,

24  stan::mcmc::sample& init\_s,

25  Model& model,

26  RNG& base\_rng,

27  const std::string& prefix,

28  const std::string& suffix,

29  std::ostream& o,

30  StartTransitionCallback& callback) {

31  for (int m = 0; m < num\_iterations; ++m) {

32  callback();

33

34  print\_progress(m, start, finish, refresh, warmup, prefix, suffix, o);

35

36  init\_s = sampler->transition(init\_s);

37

38  if ( save && ( (m % num\_thin) == 0) ) {

39  writer.write\_sample\_params(base\_rng, init\_s, \*sampler, model);

40  writer.write\_diagnostic\_params(init\_s, sampler);

41  }

42

43  }

44

45  }

46

47  }

48 }

49

50 #endif

base\_mcmc.hpp

print\_progress.hpp

mcmc\_writer.hpp

stan::mcmc::base\_mcmc::transition

virtual sample transition(sample &init\_sample)=0

stan::io::mcmc\_writer

mcmc\_writer writes out headers and samples

**Definition:** mcmc\_writer.hpp:29

stan::common::run\_markov\_chain

void run\_markov\_chain(stan::mcmc::base\_mcmc \*sampler, const int num\_iterations, const int start, const int finish, const int num\_thin, const int refresh, const bool save, const bool warmup, stan::io::mcmc\_writer< Model, SampleRecorder, DiagnosticRecorder, MessageRecorder > &writer, stan::mcmc::sample &init\_s, Model &model, RNG &base\_rng, const std::string &prefix, const std::string &suffix, std::ostream &o, StartTransitionCallback &callback)

**Definition:** run\_markov\_chain.hpp:13

stan::common::warmup

void warmup(stan::mcmc::base\_mcmc \*sampler, int num\_warmup, int num\_samples, int num\_thin, int refresh, bool save, stan::io::mcmc\_writer< Model, SampleRecorder, DiagnosticRecorder, MessageRecorder > &writer, stan::mcmc::sample &init\_s, Model &model, RNG &base\_rng, const std::string &prefix, const std::string &suffix, std::ostream &o, StartTransitionCallback &callback)

**Definition:** warmup.hpp:13

stan::mcmc::sample

**Definition:** sample.hpp:13

stan::common::print\_progress

void print\_progress(const int m, const int start, const int finish, const int refresh, const bool warmup, const std::string prefix, const std::string suffix, std::ostream &o)

**Definition:** print\_progress.hpp:13

stan::mcmc::base\_mcmc

**Definition:** base\_mcmc.hpp:13


---

[ Stan Home Page ]

*© 2011–2014,
Stan Development Team.*
